# Comparison of Plasmid Curing Efficiency across Five Lactic Acid Bacterial Species

**DOI:** 10.4014/jmb.2406.06003

**Published:** 2024-09-11

**Authors:** Chan-Hyeok Park, Haneol Yang, Seunghyun Kim, Chan-Seok Yun, Byung-Chun Jang, Yeong-Jin Hong, Doo-Sang Park

**Affiliations:** 1 Korean Collection for Type Cultures (KCTC), Korea Research Institute of Bioscience and Biotechnology (KRIBB), Jeongeup 56212, Republic of Korea; 2 BioMedical Sciences Graduate Program (BMSGP), Chonnam National University Medical School, Hwasun 58128, Republic of Korea; 3 KRIBB School of Bioscience, Korea University of Science and Technology, Daejeon 34113, Republic of Korea

**Keywords:** Lactic acid bacteria, plasmid curing, antibiotic resistance, curing agent, SNP

## Abstract

With the recent stringent criteria for antibiotic susceptibility in probiotics, the presence of antibiotic resistance genes and plasmids associated with their transfer has become a limiting factor in the approval of probiotics. The need to remove genes related to antibiotic resistance and virulence through plasmid curing for the authorization of probiotics is increasing. In this study, we investigated the curing efficiency of ethidium bromide, acridine orange, and novobiocin at different concentrations and durations in five strains of plasmid-bearing lactic acid bacteria and examined the curing characteristics in each strain. *Limosibacillus reuteri* and *Lacticaseibacillus paracasei* exhibited curing efficiencies ranging from 5% to 44% following treatment with ethidium bromide (10–50 μg/ml) for 24–72 h, while *Lactobacillus gasseri* showed the highest efficiency at 14% following treatment with 10 μg/ml novobiocin for 24 h. *Lactiplantibacillus plantarum*, which harbors two or more plasmids, demonstrated curing efficiencies ranging from 1% to 8% after an additional 72-h treatment of partially cured strains with 10 μg/ml novobiocin. Plasmid curing in strains with larger plasmids exhibited lower efficiencies and required longer durations. In strains harboring two or more plasmids, a relatively low curing efficiency with a single treatment and a high frequency of false positives, wherein recovery occurred after curing, were observed. Although certain strains exhibited altered susceptibilities to specific antibiotics after curing, these outcomes could not be attributed to the loss of antibiotic resistance genes. Furthermore, the genomic data from the cured strains revealed minimal changes throughout the genome that did not lead to gene mutations.

## Introduction

Plasmids are independently replicating DNA molecules that exist independently of chromosomal DNA [1]. They are known to possess various functions, such as F plasmid (facilitates conjugation) [2] and R plasmid (which confers resistance to antibiotics or toxins) [3]. In widely used probiotic bacteria, various types of plasmids also exist. The fertility plasmid (F plasmid) is used to transfer DNA between bacteria, enabling gene transfer between cells, which promotes genetic exchange within bacterial populations. It regulates the necessary steps in the cell conjugation process and replicates the DNA to be transferred between cells [4]. When plasmids contain antibiotic resistance genes, neighboring bacteria can acquire these genes through such mechanisms, facilitating the widespread dissemination of antibiotic resistance within bacterial populations [5].

In recent decades, bacterial antibiotic resistance has rapidly escalated. Various organizations, including the World Health Organization [6], Food and Agriculture Organization [7], Food and Drug Administration [8], and the European Food Safety Authority (EFSA) [9] are promoting awareness of this matter, which is considered a globally significant medical and public health concern [10, 11]. The heightened potential for the transfer of antibiotic resistance genes within the gut, especially when probiotics are antibiotic resistant, has prompted the proposal of safety evaluation methods for addressing antibiotic resistance gene transmission [12]. Although probiotics with exceptional functionality show improved effectiveness, they cannot be used as functional probiotics if they fail safety assessments due to antibiotic resistance. Therefore, if the probiotic plasmid DNA contains antibiotic resistance genes, the expression and transmission of these genes must be inhibited through methods that eliminate antibiotic resistance, such as plasmid curing [13]. This process is essential for passing the safety assessment and enabling registration as a functional probiotic. As a prime example, *Limosilactobacillus reuteri* ATCC 55730, which harbors antibiotic resistance genes for penicillin, tetracycline, and lincomycin, was transformed into *Lm. reuteri* DSM 17938, which had its antibiotic susceptibility restored through plasmid curing[14]. This strain is currently used in various functional health products.

Plasmid curing methods have been extensively developed, employing curing compounds such as detergents, DNA-intercalating agents, biocides, antibiotics, and plant-derived compounds [15]. In addition, methods based on plasmid incompatibility principles [16, 17], anti-plasmid systems utilizing bacteriophages [18], and clustered regularly interspaced short palindromic repeats (CRISPR)/CRISPR-associated protein-based plasmid curing systems [19] have been used [15]. In particular, the use of DNA-intercalating agents such as acridine orange (AO) and ethidium bromide (EtBr) and DNA gyrase-inhibiting drugs such as novobiocin (Nv) and coumermycin A is a common example of a widely employed traditional curing method [1, 20].

CRISPR/Cas9 technology has emerged as a highly precise and efficient method for inducing targeted genetic modifications [21]. However, applying CRISPR technology to plasmid removal in lactic acid bacteria (LAB) presents several challenges. CRISPR only cuts specific fragments of the plasmid DNA, requiring multiple edits to achieve complete removal [22]. Additionally, this includes the need for species-specific guide RNA design, the potential for off-target effects, and the requirement for sophisticated laboratory infrastructure that may not be readily accessible in all research environments [23]. In contrast, traditional mutagenesis methods such as EtBr, AO, and Nv offer the advantage of being widely applicable to various bacteria without the need for species-specific guide RNA design. These methods also allow for the simultaneous removal of multiple plasmids. Although CRISPR is known as an effective method for knocking out specific genes, challenges exist in introducing genes in LAB, and it can only be utilized if the exact target gene is known [22]. In this study, we observed a decrease in antibiotic resistance following the removal of plasmids, even though the plasmids did not contain any known antibiotic resistance genes. This suggests that complete plasmid removal may be more effective in enhancing the safety of using LAB, compared to targeting and removing specific resistance genes with CRISPR. Therefore, this study aims to evaluate the effectiveness of traditional plasmid removal methods in LAB, providing a benchmark for future research using technologies like CRISPR.

For instance of using traditional curing agents, treatment of *Escherichia coli* K12 with 25 μg/ml AO for 72 h demonstrated a 99% curing probability [24], while treatment of *E. coli* 207940 with 100 μg/ml EtBr for 48 h resulted in approximately 21% curing efficiency [25]. Nv has been used as a curing agent not only for strains belonging to Enterobacteriaceae, such as *E. coli* and *Shigella sonnei*, but also for some LAB strains, including *Lactiplantibacillus plantarum* [26, 27], and excellent curing efficiencies ranging from 4.0% to 90% have been reported.

However, the concentration of the agents and treatment time for plasmid curing vary, and no protocol has been precisely defined. In preliminary studies wherein LAB strains carrying plasmids are treated with varying concentrations of curing agents, extremely low curing efficiencies or recovery of plasmids in the cured strains as false-positive results have been verified (Fig. S1). These problems require the reestablishment of curing methods applicable to LAB.

In this study, we investigated the curing efficiencies of widely used curing agents, namely EtBr, AO, and Nv, in 10 strains of LAB belonging to five different species, whose plasmid types, sizes, G+C contents, and coding sequences were elucidated through whole-genome analysis. We compared the curing efficiency in each strain and examined whether antibiotic susceptibility changed or genetic variations occurred after plasmid curing.

## Materials and Methods

### Strains and Culture Medium

The LAB strains were obtained from the Bio R&D Product program (https://biorp.kribb.re.kr/). The bacterial strains were cultivated in de Man, Rogosa, and Sharpe (MRS) media (BD, USA) under anaerobic conditions at 37°C for 24–48 h. The names, numbers of plasmids, plasmid sizes, and G+C content of the strains used in this study are listed in Table 1. Whole-genome sequences before and after plasmid curing have been deposited in the National Center for Biotechnology Information database (Table S1).

### Plasmid Curing

**Fig. 1:**
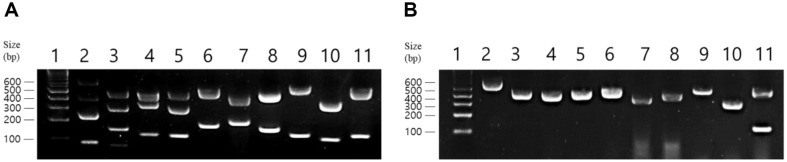
Agarose gel electrophoresis of PCR-amplified DNA fragments from wild-type and cured strains. (**A**) PCR-amplified products from the chromosomes and plasmids of wild-type LAB strains. (**B**) PCR-amplified products from the chromosomes and plasmids of plasmid-cured strains. Lane 1, 1 kb plus DNA ladder; lane 2, *L. plantarum* DS1989; lane 3, *L. plantarum* DS0815; lane 4, *L. plantarum* DS1902; lane 5, *L. plantarum* DS1073; lane 6, *Lm. reuteri* DS0354; lane 7, *Lm. reuteri* DS0384; lane 8, *L. gasseri* DS2831; lane 9, *Lc. paracasei* DS0725; lane 10, *Lc. paracasei* DS2766; lane 11, *B. longum* DS1566. Curing of *B. longum* DS1566 was not achieved.

Plasmid curing was performed with EtBr, AO, and Nv as curing agents at concentrations of 10 μg/ml and 50 μg/ml. Treatment with the curing agent was performed for 24, 48, and 72 h until sufficient curing data were obtained. When complete curing was not achieved in the primary curing reaction, secondary curing was conducted using partially cured strains. For secondary curing, the reaction was conducted for up to 72 h using the most effective agent concentration determined from the primary reaction. The LAB strain (10^8^ colony-forming unit CFU/ml) was inoculated (2% v/v) into the MRS broth containing the curing agent and incubated statically in an anaerobic chamber at 37°C for 24, 48, and 72 h. At each time point, the bacterial solution was spread onto MRS agar plates and incubated for 2–3 days. Colonies obtained from these plates were selected. To confirm the presence or absence of plasmids, colony PCR was performed by picking each colony using a toothpick and adding 10 μl of PCR Master Mix (Bioneer, Republic of Korea) containing 3.2 pmol of each primer, and distilled water was added to obtain a final volume of 20 μl. Specific primers for chromosomes and plasmids in each LAB strain were designed based on whole-genome sequencing data. The PCR conditions were set as follows: 35 cycles of denaturation at 94°C for 30 sec, annealing at 59°C for 30 sec, and extension at 72°C for 30 sec. The PCR products were analyzed via electrophoresis on 1.5% agarose gels. The amplified fragments were of different sizes, allowing simultaneous verification of the chromosome and plasmid products in a single electrophoresis run (Fig. 1, Table S2). Plasmid curing was successfully achieved if the PCR results showed amplification of the chromosomal DNA but not of the plasmid DNA. The plasmid-cured cells were then suspended in phosphate-buffered saline, spread onto MRS agar plates, and incubated. The resulting colonies were subjected to the same colony PCR procedure to verify plasmid recovery.

### Statistical Analysis

Statistical analyses were conducted to evaluate the significance of differences in plasmid removal rates among different agents and concentrations. An analysis of variance (ANOVA) was performed to determine if there were statistically significant differences between groups. Fisher’s exact test and chi-square test were used to calculate the probability values for categorical data. A *p* < 0.05 was considered statistically significant. All statistical analyses were conducted using SPSS (version 25.0) [28] and R (version 4.0.2) ( https://www.R-project.org/).

### Principal Component Analysis (PCA)

Principal Component Analysis (PCA) was performed to reduce the dimensionality of the dataset and to identify patterns and relationships between different strains and agents. The data matrix consisted of plasmid removal rates for different strains treated with various agents at two concentrations (10 μg/ml and 50 μg/ml). Prior to PCA, the data were standardized to ensure each variable contributed equally to the analysis. PCA was conducted using the sklearn library in Python (version 0.24.2) [29], and the results were visualized in scatter plots. The first two principal components were used to create a two-dimensional plot where each point represents a strain-agent combination, colored by the removal rate.

### DNA Extraction and Genomic Analysis

Bacterial genomic DNA was extracted using the phenol:chloroform:isoamyl alcohol method [30], and whole-genome sequencing was performed using a PacBio RS II platform at Macrogen Inc. (Republic of Korea). The sequencing data were assembled *de novo* using SPAdes (version 3.13.0). Genome annotation was performed using Prokka (version 1.14.6), and genome analysis was performed using ISfinder, NCBI Background Reference Gene DB, UnitProtKB DB, and HMM DB. Genomic G + C content was calculated by analyzing the draft genome [31]. For cured bacteria, whole-genome resequencing was performed on an Illumina platform (Macrogen). After mapping the reads, variants (insertions, deletions, and single nucleotide polymorphisms [SNPs]) were identified using SAMTools and compared to the genome of wild-type bacteria. In addition, the presence of genes related to antibiotic resistance or virulence in their chromosomes and plasmids in the LAB strains used in the study was determined using Resfinder (version 4.5.0) and Virulence Finder (version 2.0), provided by the Center for Genomic Epidemiology (http://www.genomicepidemiology.org/) [32, 33].

To confirm the copy number of the plasmid, RNA from the strains was extracted using the RNeasy extraction kit (Qiagen, Germany), and 1 μg of RNA was synthesized into cDNA using the iScript cDNA Synthesis Kit (Bio-Rad Laboratories, USA). Quantitative real time polymerase chain reaction (qRT-PCR) was performed on a CFX-96 real-time PCR system (Bio-Rad) as previously described [34] using self-designed primers (Table S2). The 2^-ΔΔCT^ method was employed to calculate the copy number of the plasmid per chromosome in each strain [35].

### Analysis of Variation in Antibiotic Susceptibility for Plasmid-Cured LAB

To determine and compare the antibiotic susceptibility of each *Lactobacillus* strain before and after plasmid curing, minimum inhibitory concentration (MIC) test strips (Liofilchem s.r.l., Italy) for ampicillin, vancomycin, gentamicin, kanamycin, streptomycin, erythromycin, clindamycin, tetracycline, and chloramphenicol were used according to the manufacturer's instructions. Bacteria cultured in MRS broth for 16–24 h were adjusted to 10^8^ CFU/ml. A 100 μl aliquot of this suspension was spread onto MRS agar plates. Antibiotic strips were then placed on the plates, and they were incubated in an anaerobic chamber for 48 h. MIC values were determined by reading the intersection of the test strip and the lower part of the ellipse-shaped growth inhibition zone.

## Result

### Plasmid Curing

**Fig. 2:**
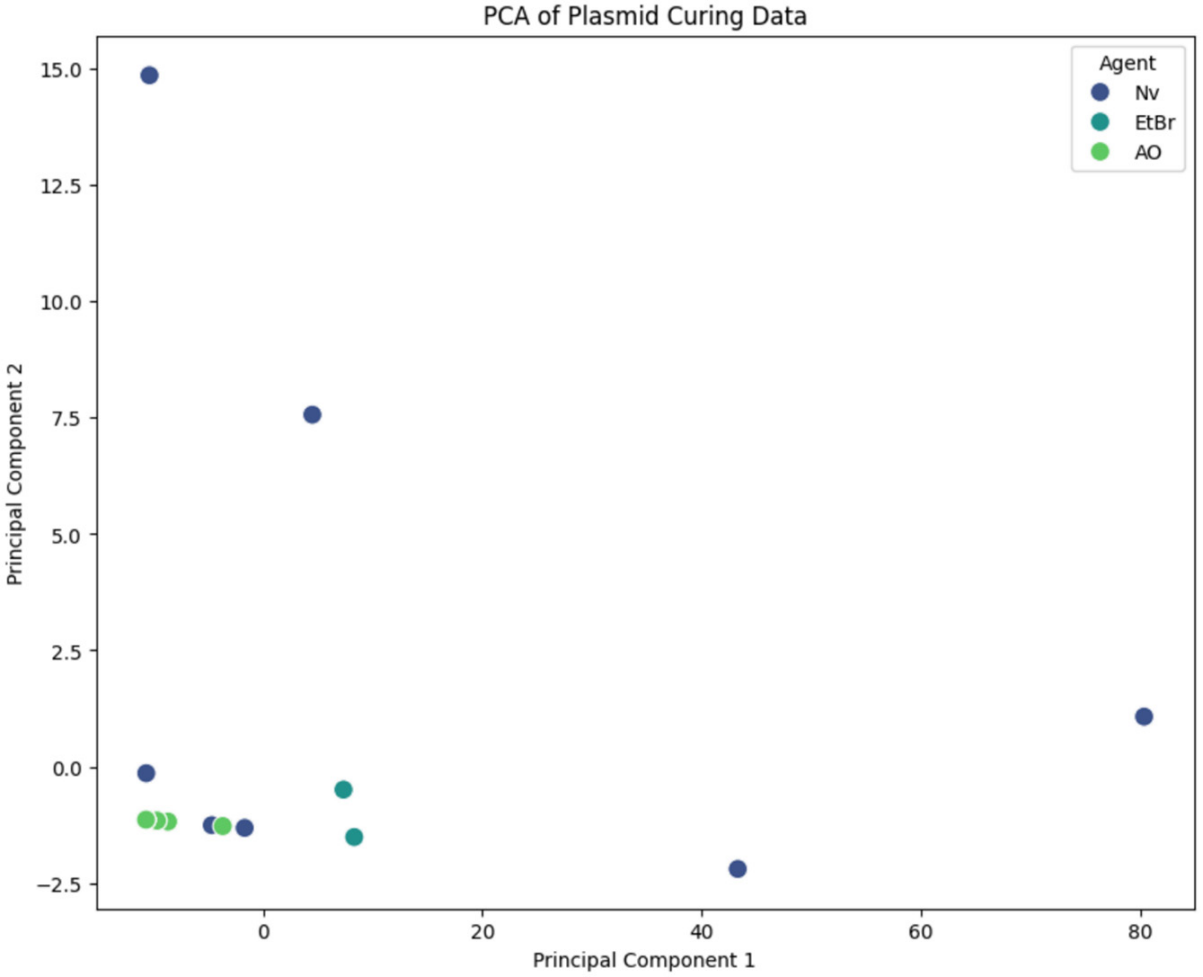
Principal Component Analysis (PCA) of plasmid curing data. The scatter plot shows the first and second principal components (PC1 and PC2) based on the plasmid curing rates of various strains treated with agents at 10 μg/ml and 50 μg/ml concentrations. Each point represents a strain-agent combination, color-coded according to the curing rate (%), visually illustrating patterns and relationships.

The curing efficiencies in *Lm. reuteri* strains DS0354 and DS0384 were 29% and 45%, respectively, after treatment with 10 μg/ml EtBr for 24 h and ranged from 3% to 10% after treatment with AO, whereas no growth was observed with 50 μg/ml EtBr or with Nv (Table 2). In the case of *Lactobacillus gasseri* DS2831, treatment with 10 μg/ml Nv for 24 h had a curing efficiency of 15%; however, DS2831 did not grow in a medium containing 50 μg/ml Nv, and curing was not achieved with AO or EtBr. Curing *Lc. paracasei* DS0725 had an efficiency of 5% after treatment with 50 μg/ml EtBr for 72 h but was not successful with AO treatment. For *Lc. paracasei* DS2766, a curing efficiency of 22% was achieved when cultured with 50 μg/ml EtBr or AO for 48 h. In both the DS0725 and DS2766 strains, bacterial growth was not observed following Nv treatment. *Bifidobacterium longum* DS1566 did not grow at any concentration of the tested agents, even when the concentration was reduced to 0.1 μg/ml, confirming that plasmid curing was not achievable. For *Lp. plantarum* strains carrying two or more plasmids, the plasmids could not be completely cured after 72 h of cultivation in a medium containing each agent (Table 3). However, a curing efficiency of 1-8% was observed upon retreatment of the partially cured strains with 10 μg/ml Nv for 72 h, which was found to be the most effective concentration for plasmid curing in *Lp. plantarum* strains (Table 2). Additionally, when comparing the curing efficiency based on plasmid size and copy number, it was observed in *Lp. plantarum* strains that even with plasmid sizes ranging from 2.4 kb to 66.8 kb, the curing efficiency sometimes increased when the copy number was lower, regardless of the larger plasmid size (Table 4). However, even when the copy number ranged from 1.9 to 142.2, there were cases where differences in curing efficiency were observed despite the small difference in copy number, and this was not consistent in all cases. In the case of *Lc. paracasei*, when comparing plasmids with the same number but with a size difference of about 10 times, the curing efficiency was lower for larger plasmid sizes (Table 4). Principal Component Analysis (PCA) was performed to identify patterns and relationships between various strains and agents concerning plasmid curing efficiency. The data matrix consisted of plasmid removal rates for strains treated with 10 μg/ml and 50 μg/ml concentrations of the agents. The PCA results are visualized in Fig. 2, where each point represents a strain-agent combination, color-coded according to plasmid removal efficiency. The first and second principal components account for the majority of the variance in the data, showing distinct clustering of strain-agent combinations based on their plasmid curing efficiency. According to the positioning of each cluster, AO and EtBr exhibited low curing efficiency, while Nv demonstrated high curing efficiency, allowing for a clear differentiation of curing efficiency among the agents.

### Variation in Antibiotic Susceptibility for Plasmid-Cured LAB

After plasmid curing, three *Lp. plantarum* strains (DS1989, DS1902, and DS1073) showed a decrease in the MIC values for gentamicin by more than half (Table 5). Susceptibility of *Lp. plantarum* (DS1902 and DS1073) to tetracycline decreased up to 1/10-fold after curing, whereas that of *Lm. reuteri* DS0384 increased. *Lm. reuteri* DS0384 and DS0354 and *L. gasseri* DS2831 had decreased MIC values for streptomycin. In terms of susceptibility to chloramphenicol, four strains (*Lp. plantarum* DS1902, *L. gasseri* DS2831, and *Lc. paracasei* DS2766 and DS0725) had MIC values that decreased by half, while *Lm. reuteri* DS0384 had an increased MIC value, as listed in Table 5.

### Genomic Variation of Plasmid-Cured LAB

Plasmid-cured strains, including *Lp. plantarum* DS1902 and DS1073, *Lm. reuteri* DS0384, *Lc. paracasei* DS0725, and *L. gasseri* DS2831, were sequenced again to investigate the presence of mutations in the genome induced by treatment with a curing agent. *Lm. reuteri* DS0384 showed the greatest number of variations, with 40 different variants due to new base insertions (Table 6). Other strains exhibited 2 to 14 variants, with single nucleotide polymorphisms (SNPs) and new base insertions occurring frequently, while deletions were relatively rare.

## Discussion

Research on curing LAB has been reported less frequently than that on pathogenic strains or species showing multi-drug resistance. Cases of plasmid curing in LAB using 100 μg/ml of AO [36], 2–10 μg/ml of acriflavine [37, 38], 8–10 μg/ml of EtBr [26], and 0.1–40 μg/ml of Nv [26, 27, 39] are representative examples. In this study, three curing agents (EtBr, AO, and Nv) were applied to 10 LAB strains from five species that are widely used as probiotics, whose plasmid numbers, sizes, and gene contents were determined through whole-genome analysis. This study aimed to reassess the differences in susceptibility to each curing agent among species and strains and to evaluate the efficiency of curing. Additionally, this study aimed to explore useful approaches for achieving complete curing of strains harboring multiple plasmids.

The DNA-intercalating agents EtBr and AO, and the DNA gyrase inhibitor Nv were used as curing agents at concentrations ranging from 10–50 μg/ml. No microbial growth was observed in the medium containing Nv for *Lm. reuteri* (DS0354 and DS0384) and *Lc. paracasei* (DS0725 and DS2766). Interestingly, *B. longum* DS1566 did not exhibit microbial growth in the presence of curing agents at any concentration (Table 2), even when diluted to 1/100. However, other strains of *B. longum* (AM54 and DS4107) grown in the laboratory were able to grow in MRS broth containing all curing agents (data not shown), suggesting that the phenomenon observed in the DS1566 strain is specific to it. Analysis of whole-genome sequencing data revealed that the DS1566 strain harbors the serine/threonine-protein kinase toxin HipA gene on its plasmid, which is distinct from the other LAB strains used in this study. HipA, derived from *E. coli*, acts as a toxic gene and is expressed in response to external factors such as antibiotics or stressors [40]. Expression of this gene inhibits the growth of the strain, and this effect is sometimes counteracted by an effective antidote, HipB [41, 42]. Ultimately, the sensitivity of LAB strains to each curing agent may vary, including in specific cases such as *B. longum* DS1566. Further accumulation of cases is necessary to determine the plasmid-curing characteristics of each species.

Following plasmid curing, 10 μg/ml EtBr exhibited the highest curing efficiency at 29–40% in *Lm. reuteri*. For *L. gasseri*, a curing efficiency of 15% was determined with 10 μg/ml Nv. For *Lc. paracasei*, relatively high concentrations of EtBr and AO exhibited superior curing efficiency after 48–72 h of treatment (Table 2). During the plasmid curing of *Lp. plantarum*, complete elimination of all plasmids was not achieved even after treatment with the three agents for up to 72 h. Additionally, despite the absence of amplification products for all plasmids via PCR analysis, false-positive instances were observed upon cultivation and repeated PCR (Fig. S1). Based on plasmid 1, which is the most easily cured plasmid among two or more plasmids, the curing efficiency of 10 μg/ml Nv in *L. plantarum* DS1989, 0815, and 1073 was 16%, 95%, and 56%, respectively. However, plasmid 1 was not cured even after 72 h of treatment in *L. plantarum* DS1902 (Table 3). These curing efficiencies are inconsistent with previously reported cases wherein 94–100% curing was achieved via treatment with 0.125–0.25 μg/ml Nv [26]. In studies that report high curing efficiency, successful curing was confirmed even if only one of the plasmids, among those ranging from 5–16 plasmids and varying in size from 2–68 kb, underwent curing [26]. Therefore, it is challenging to consider cases of partial curing success as typical examples of plasmid curing in *L. plantarum*. In this study, we examined the curing efficiency of 0.2–1 μg/ml Nv treatment in *Lp. plantarum* strains, and Nv at a concentration of 10 μg/ml was deemed more efficient as it led to curing in all *Lp. plantarum* strains. Furthermore, an additional treatment of 10 μg/ml Nv for 72 h was conducted on strains harboring two to three plasmids to achieve complete curing of all plasmids. Through this process, complete curing was achieved in only 1–8% of the cases, indicating a very low success rate (Table 2).

During plasmid curing in *Lp. plantarum* strains harboring two or more plasmids, strains with plasmid sizes of 48–66 kb showed efficiencies ranging from 9–95%, indicating that they were generally more easily cured than those with plasmids smaller than 20 kb (Table 4). However, the maximum curing efficiency for the 6.2 kb plasmid of *Lc. paracasei* DS2766 and the 66.7 kb plasmid of *Lc. paracasei* DS0725 was 22% after 48 hours and 5% after 72 h in contrast to *Lp. plantarum*. This suggests that other factors may influence plasmid curing efficiency. For example, previous studies have shown that the cell growth phase can affect plasmid removal [43]. Plasmids are more stably maintained when cells are in the early growth phase. However, as cells progress to the mid-growth phase and cell division becomes more active, plasmid removal can be enhanced. The plasmid copy numbers in *Lp. plantarum* strains varied widely, ranging from approximately 1.9 to 142.2 copies, while the G+C content ranged from 36.0% to 40.5%, indicating a relatively similar range. However, these two factors did not significantly affect plasmid curing (Table 4).

Additionally, the genomes of 10 LAB strains from 5 species used in this study were analyzed using Resfinder and Virulence Finder. The analysis revealed no genes related to antibiotic resistance or virulence in the chromosomes or plasmids. However, some strains exhibited resistance that exceeded the antibiotic susceptibility guidelines proposed by the EFSA (Table S3). A comparison of antibiotic susceptibility before and after plasmid curing revealed a decrease in resistance to gentamicin by 1/2- and 1/4-fold in *Lp. plantarum* (Table 5). In the case of *Lm. reuteri*, both strains showed a tendency toward decreased resistance to streptomycin, while *Lc. paracasei* showed a tendency toward decreased resistance to chloramphenicol. However, for the cured strain of *Lm. reuteri* DS0384, tetracycline resistance increased more than six fold. This is in contrast to the results for *Lp. plantarum* DS1703 and DS1902 strains, wherein the resistance decreased by 1/6-fold (Table 5). These results indicate the involvement of factors other than the currently known antibiotic resistance-related genes and their mechanisms, which remain to be elucidated. In such cases, the plasmid removal process can cause stress to the cells, potentially leading to genetic recombination or mutations, which may increase antibiotic resistance [44, 45].

The curing agents AO and EtBr, which are DNA-intercalating agents, are well-known mutagens that induce genetic mutations upon prolonged exposure[46]. Sequencing and variant calling of the five cured strains revealed that *Lm. reuteri* DS0384, which underwent curing with 10 μg/mL EtBr for 24 h, had the highest number of variants among the strains, having 40 SNP mutations (Table 6). However, for *Lc. paracasei* DS0725, which underwent curing with a higher concentration of EtBr at 50 μg/ml, only three mutations were observed despite treatment for 72 h. In addition, in strains cured using Nv, which induces changes in plasmid topology by acting as a DNA gyrase inhibitor, 2–14 variants were detected. However, variations of 2–40 nucleotides are considered to occur in LAB, which typically has an average genome size of approximately 2.6 GB. Therefore, it is presumed that mutations induced by treatment with curing agents do not significantly affect the functional characteristics of the strains, unless they alter the expression of important genes.

In conclusion, when considering the curing probability with the three agents across different LAB species, it is more efficient to use Nv for curing in *Lp. plantarum* and *L. gasseri*. For *Lm. reuteri*, EtBr is more effective, while *Lc. paracasei* can benefit from curing with EtBr and AO to increase the success rate of plasmid removal. Additionally, when the plasmid size is large, higher agent concentrations and longer treatment times are required compared to smaller plasmids. Moreover, if there are multiple plasmids, achieving complete curing may require undergoing the curing process twice. Our study demonstrates that traditional mutagenic agents such as EtBr, AO, and Nv can effectively remove plasmids from various LAB strains, achieving up to 45% curing efficiency under optimal conditions. These results are important as they provide a benchmark for evaluating technologies like CRISPR. While CRISPR offers precise and targeted genetic modifications, traditional methods are extremely useful in initial screenings and broad applications due to their wide applicability and relative simplicity. Additionally, genome analysis after plasmid removal showed that off-target effects were minimized, highlighting the specificity of traditional mutagenic agents when used under controlled conditions. This offers a practical alternative to using CRISPR technology for ensuring the safety of probiotic strains.

## Supplemental Materials

Supplementary data for this paper are available on-line only at http://jmb.or.kr.



## Figures and Tables

**Table 1:** Scientific names, strain numbers, number of plasmids, plasmid size, and G+C contents of the LAB used in this study.

Scientific name	Strain	Plasmids	Accession number for wild-type strain	Plasmid size (bp)	G+C content (%)
*Lactiplantibacillus plantarum*	DS1989	Plasmid 1 Plasmid 2 Plasmid 3	CP146869 CP146870 CP146871	50,933 48,573 8,694	38.9 39.0 36.0
	DS0815	Plasmid 1 Plasmid 2 Plasmid 3	CP146873 CP146874 CP146875	66,660 39,431 6,156	39.3 40.5 37.4
	DS1902	Plasmid 1 Plasmid 2	CP146866 CP146867	7,845 2,410	36.9 38.2
	DS1073	Plasmid 1 Plasmid 2	CP147893 CP147894	50,512 19,584	39.0 40.5
*Limosilactobacillus reuteri*	DS0354	Plasmid 1	CP146877	19,051	36.9
	DS0384	Plasmid 1	CP090314	20,351	37.1
*Lactobacillus gasseri*	DS2831	Plasmid 1	CP146881	49,996	36.0
*Lacticaseibacillus paracasei*	DS0725	Plasmid 1	CP151182	66,795	43.8
	DS2766	Plasmid 1	CP146879	6,196	38.9
*Bifidobacterium longum*	DS1566	Plasmid 1	CP146883	193,392	57.2

**Table 2:** Plasmid curing efficiency in the tested strains.

Scientific name	Strain	Agent / (μg/ml)	Incubation time (h)	Curing rate (%)
*Lactiplantibacillus plantarum*	DS1989	Nv / 10	72+72	1/96 (1%)
*Lactiplantibacillus plantarum*	DS0815	Nv / 10	72+72	1/96 (1%)
*Lactiplantibacillus plantarum*	DS1902	Nv / 10	72+72	2/96 (2%)
*Lactiplantibacillus plantarum*	DS1073	Nv / 10	72+72	8/96 (8%)
*Limosilactobacillus reuteri*	DS0354	EtBr / 10	24	28/96 (29%)
		EtBr / 50	-	
		AO / 10	24	3/96 (3%)
		AO / 50	24	10/96 (10%)
		Nv / 10	-	
		Nv / 50	-	
*Limosilactobacillus reuteri*	DS0384	EtBr / 10	24	43/96 (45%)
		EtBr / 50	-	
		AO / 10	24	4/96 (4%)
		AO / 50	-	
		Nv / 10	-	
		Nv / 50	-	
*Lactobacillus gasseri*	DS2831	EtBr / 10	24	0/96
		EtBr / 50	24	0/96
		AO / 10	24	0/96
		AO / 50	24	0/96
		Nv / 10	24	14/96 (15%)
		Nv / 50	-	
*Lacticaseibacillus paracasei*	DS0725	EtBr / 10	72	0/96
		EtBr / 50	72	5/96 (5%)
		AO / 10	72	0/96
		AO / 50	72	0/96
		Nv / 10	-	
		Nv / 50	-	
*Lacticaseibacillus paracasei*	DS2766	EtBr / 10	24	4/96 (4%)
		EtBr / 50	24	4/96 (4%)
		EtBr / 10	48	7/96 (7%)
		EtBr / 50	48	21/96 (22%)
		AO / 10	48	17/96 (18%)
		AO / 50	48	21/96 (22%)
		Nv / 10	-	
		Nv / 50	-	
*Bifidobacterium longum*	DS1566	EtBr / 10	-	-
		EtBr / 50	-	-
		AO / 10	-	-
		AO / 50	-	-
		Nv / 10	-	-
		Nv / 50	-	-

–, the strain does not grow in medium containing a curing agent. EtBr, Ethidium bromide; AO, Acridine orange; Nv, Novobiocin.

**Table 3:** Curing in LAB strains harboring more than two plasmids.

Scientific name	Strain	Agent / (μg/ml)	Incubation time (h)	Plasmid 1 cured colonies	Plasmid 2 cured colonies	Plasmid 3 cured colonies	Plasmid 1&2 cured colonies	Plasmid 1&3 cured colonies	Plasmid 2&3 cured colonies	Plasmid 1&2&3 cured colonies
*Lactiplantibacillus plantarum*	DS1989	Nv / 10	72	15/96 (16%)	9/96 (9%)	1/96 (1%)	1/96 (1%)	0/96	0/96	0/96
		Nv / 50	72	0/96	0/96	0/96	0/96	0/96	0/96	0/96
		EtBr / 50	72	1/96 (1%)	0/96	0/96	0/96	0/96	0/96	0/96
		EtBr/ 10	72	1/96 (1%)	0/96	0/96	0/96	0/96	0/96	0/96
		AO / 10	72	2/96 (2%)	0/96	0/96	0/96	0/96	0/96	0/96
		AO / 50	72	2/96 (2%)	0/96	0/96	0/96	0/96	0/96	0/96
*Lactiplantibacillus plantarum*	DS0815	Nv / 10	72	91/96 (95%)	4/96 (4%)	4/96 (4%)	4/96 (4%)	4/96 (4%)	0/96	0/96
		Nv / 50	72	6/6 (100%)	0/6	0/6	0/6	0/6	0/6	0/6
		EtBr / 10	72	9/96 (9%)	0/96	0/96	0/96	0/96	0/96	0/96
		EtBr/ 50	72	18/22 (82%)	1/22 (5%)	0/22	0/22	0/22	0/22	0/22
		AO / 10	72	9/96 (9%)	0/96	0/96	0/96	0/96	0/96	0/96
		AO / 50	72	7/96 (7%)	0/96	0/96	0/96	0/96	0/96	0/96
*Lactiplantibacillus plantarum*	DS1073	Nv / 10	72	54/96 (56%)	0/96	-	0/96	-	-	-
		Nv / 50	72	9/96 (9%)	0/96	-	0/96	-	-	-
		EtBr / 10	72	1/96 (1%)	0/96	-	0/96	-	-	-
		EtBr / 50	72	19/96 (20%)	0/96	-	0/96	-	-	-
		AO / 10	72	1/96 (1%)	0/96	-	0/96	-	-	-
		AO / 50	72	0/96	0/96	-	0/96	-	-	-
*Lactiplantibacillus plantarum*	DS1902	Nv / 10	72	0/96	16/96 (17%)	-	0/96	-	-	-
		Nv / 50	72	0/96	1/96 (1%)	-	0/96	-	-	-
		EtBr / 10	72	0/96	0/96	-	0/96	-	-	-
		EtBr / 50	72	0/96	0/96	-	0/96	-	-	-
		AO / 10	72	0/96	0/96	-	0/96	-	-	-
		AO / 50	72	0/96	0/96	-	0/96	-	-	-

–, the strain does not possess plasmid 3. EtBr, Ethidium bromide; AO, Acridine orange; Nv, Novobiocin.

**Table 4:** Plasmid curing efficiency based on plasmid size.

Strain	Number of plasmids	Plasmid size (bp)	Copy number	G+C content (%)	Agent / (μg/ml)	Incubation time (h)	Cured colonies	Curing (%)
DS1989	3	50,933	38.9	38.9	EtBr/ 10	72	1/96	1
					EtBr / 50	72	1/96	1
					AO / 10	72	2/96	2
					AO / 50	72	2/96	2
					Nv / 10	72	15/96	15
		48,573	121.5	39.0	Nv / 10	72	9/96	9
		8,694	142.2	36.0	Nv / 10	72	1/96	1
DS0815	3	66,660	4.3	39.3	EtBr/ 10	72	9/96	9
					EtBr / 50	72	18/22	82
					AO / 10	72	9/96	9
					AO / 50	72	7/96	7
					Nv / 10	72	91/96	95
					Nv / 50	72	6/6	100
		39,431	3.6	40.5	EtBr / 50	72	1/22	5
					Nv / 10	72	4/96	4
		6,156	8.6	37.4	Nv / 10	72	4/96	4
DS1073	2	50,512	1.9	39.0	EtBr / 10	72	1/96	1
					EtBr / 50	72	19/96	20
					AO / 10	72	1/96	1
					Nv / 10	72	54/96	56
					Nv / 50	72	9/96	9
		19,584	4.2	40.5	ALL	72	0/96	0
DS1902	2	7,845	3.4	36.9	ALL	72	0/96	0
		2,410	9.8	38.2	Nv / 10	72	16/96	17
					Nv / 50	72	1/96	1
DS0725	1	66,795		43.8	EtBr / 50	72	5/96	5
DS2766	1	6,196		38.9	EtBr / 10	24	4/96	4
					EtBr / 50	24	4/96	4
					EtBr / 10	48	7/96	7
					EtBr / 50	48	21/96	22
					AO / 10	48	17/96	18
					AO / 50	48	21/96	22

EtBr, Ethidium bromide; AO, Acridine orange; Nv, Novobiocin.

**Table 5:** Changes in the antibiotic susceptibility of each strain after plasmid curing.

Strain Antibiotic	DS1989 *Lp plantarum*		DS0815 *Lp plantarum*		DS1073 *Lp plantarum*		DS1902 *Lp plantarum*		DS0354 *Lm. reuteri*		DS0384 *Lm. reuteri*		DS2831 *L. gasseri*		DS0725 *Lc. paracasei*		DS2766 *Lc. paracasei*	
	WT	C	WT	C	WT	C	WT	C	WT	C	WT	C	WT	C	WT	C	WT	C
Ampicillin	0.125	0.125	0.125	0.064	0.94	0.94	0.19	0.19	3	4	**4**	**8**	0.38	0.25	0.75	0.75	1	1
Vancomycin	-	-	-	-	-	-	-	-	-	-	-	-	1.5	1	-	-	-	-
Gentamycin	**64**	**24**	48	64	**100**	**24**	**48**	**24**	**12**	**6**	**16**	**12**	32	32	24	24	64	64
Kanamycin	256	256	256	256	256	256	256	256	256	256	256	256	128	256	**256**	**96**	256	256
Streptomycin	-	-	-	-	-	-	-	-	**96**	**48**	**128**	**96**	16	12	32	48	256	256
Erythromycin	1	1	1	0.75	1.5	1	1	1	0.75	0.5	0.5	1	0.5	0.38	0.38	0.38	0.5	0.5
Clindamycin	1	1	0.75	0.75	0.75	0.75	1	1	0.125	0.047	0.094	0.125	8	12	0.047	0.94	0.38	0.38
Tetracycline	16	16	4	4	**12**	**2**	**64**	**6**	16	16	**12**	**96**	1.5	1.5	0.38	0.5	0.75	0.5
Chloramphenicol	8	8	4	4	6	6	**12**	**6**	3	4	2	4	**6**	**3**	**8**	**4**	**12**	**6**

WT, wild-type strain; C, plasmid-cured strain. Instances where antibiotic resistance has significantly decreased or increased are highlighted in bold.

**Table 6:** Analysis of genomic variation induced by treatment with the curing agents.

Strain	Scientific name	Number of SNPs	Number of insertions	Number of deletions	Variants
DS1073	*Lactiplantibacillus plantarum*	6	7	1	14
DS1902	*Lactiplantibacillus plantarum*	2	0	0	2
DS0384	*Limosilactobacillus reuteri*	0	40	0	40
DS0725	*Lacticaseibacillus paracasei*	3	0	0	3
DS2831	*Lactobacillus gasseri*	4	5	1	10
