# Supplementary material for: Comparison of Plasmid Curing Efficiency across Five Lactic Acid Bacterial Species
Source: J Microbiol Biotechnol. 2024 Sep 11;34(11):2385–95. doi: 10.4014/jmb.2406.06003 (PMC11637826; doi:10.4014/jmb.2406.06003)

J. Microbiol. Biotechnol. 2024. 34(11): 2385–2395

https://doi.org/10.4014/jmb.2406.06003

Supplementary Tables and Figure

Comparison of plasmid curing efficiency across five Lactic acid bacterial species

Chan-Hyeok Park[1, 2], Haneol Yang[1], Seunghyun Kim[1, 2], Chan-Seok Yun[1], Byung-Chun Jang[1, 2], Yeong-Jin Hong[2*], and Doo-Sang Park[1, 3*]

[1]Korean Collection for Type Cultures (KCTC), Korea Research Institute of Bioscience and Biotechnology (KRIBB), Jeongeup, 56212, Republic of Korea

[2] BioMedical Sciences Graduate Program (BMSGP), Chonnam National University Medical School, Hwasun, 58128, Republic of Korea

[3]KRIBB School of Bioscience, Korea University of Science and Technology, Daejeon, 34113, Republic of Korea

*** Corresponding authors:**
Doo-Sang Park, Yeong-Jin Hong

E-mail: dspark@kribb.re.kr

Table S1. Information about the LAB strains used in the study.

Scientific name	Strain	Deposition No.	Accession No. for wild-type strain	Accession No. for cured strain	Source of isolation	Year of isolation	Country of isolation
Lactiplantibacillus plantarum	DS1989	BP1884041	CP146868<br>CP146869<br>CP146870<br>CP146871		Kimchi	2018	Republic of Korea.
Lactiplantibacillus plantarum	DS0815	BP1883382	CP146872<br>CP146873<br>CP146874<br>CP146875		Fermented pork	2017	Republic of Korea.
Lactiplantibacillus plantarum	DS1902	BP1883956	CP146865<br>CP146866<br>CP146867	CP147888	Kimchi	2018	Republic of Korea.
Lactiplantibacillus plantarum	DS1073	BP1883494	CP147892<br>CP147893<br>CP147894	CP147887	Human feces	2017	Republic of Korea.
Limosilactobacillus reuteri	DS0354	BP1883123	CP146876<br>CP146877		Human feces	2017	Republic of Korea.
Limosilactobacillus reuteri	DS0384	14164BP	CP090313<br>CP090314	CP147889	Human feces	2017	Republic of Korea.
Lactobacillus gasseri	DS2831	BP1884703	CP146880<br>CP146881	CP147891	Human feces	2019	Republic of Korea.
Lacticaseibacillus paracasei	DS0725	15516BP	CP151181<br>CP151182	CP147890	Human feces	2017	Republic of Korea.
Lacticaseibacillus paracasei	DS2766	BP1884639	CP146878<br>CP146879		Human feces	2019	Republic of Korea.
Bifidobacterium longum	DS1566	BP1883610	CP146882<br>CP146883		Human feces	2018	Republic of Korea.

Table S2. Self-designed primers used in PCR.

Strain	Forward primers	Reverse primers	Size (bp)
DS1989	5’-CCGTCAATGGACCCAAGACA-3’	5′-CATGACGCCGAGGTCGAATA-3′	661
	5’-GCTCCCAAGAGTGTAGAAGA-3’	5′-TGCCGATTCTTCGGTAGTTCC-3′	404
	5’-AGGTGTGATGGGCCTCAGTA-3’	5′-TGCATCCCGCCTGATAACTG-3′	240
	5′-TTAACGATGGCACCGCATGA-3′	5′-TGACTCCGGGGTGCTATTTTC-3′	84
DS0815	5′-GGGTGGTGCCTCCGTTATAG-3′	5′-TGCATTACGCCAAGACCGAT-3′	453
	5′-ACGCTTATAATGGCGACTGGA-3′	5′-CTAGACATCCTCGGCAACAT-3′	299
	5′-GTGGGATTACACAGAACGGC-3′	5′-TCTAACCGTTGCCGATTCCC-3′	155
	5′-GTGGGATTACACAGAACGGC-3′	5′-TCTAACCGTTGCCGATTCCC-3′	73
DS1902	5′-GCTGAATTGACCCACATCGC-3′	5′-TAACCAGTCAGGGTCGGCTA–3′	441
	5’-AATACCGGCTGATAGGCAACC-3’	5′-CGTCTGTATCGTCGCTTTGC-3′	335
	5′-TCGGCACTAGCTTGCAAAGA-3′	5′-GCACAAGCAGAATGGGCAAA-3′	125
DS1073	5′-TCAACCTACCGACGCTTGAC-3′	5′-GGCACCTAGTGGATTGACCC-3′	461
	5’-GAAATATGGCTATGCGCGGG-3’	5′-CTAATTGGCCAGTCGGCGTA-3′	308
	5′-CGCAAGTGGCTGATCAAGGA-3′	5′-ATTTCCAGCATGCCACCCA-3′	122
DS0354	5′-ACCAAACATTAACCGGGCCA-3′	5′-ATTATCAGCGGCAGGGTCTG-3′	506
	5′-TTGGACAATGGGACATGCGA-3′	5′-TGCCGCCATTGATTCCTTCT-3′	187
DS0384	5′-CTATGGCAGCCCTTAGTCGG-3′	5′-TCAATATGGTCCGCCACTCG-3′	397
	5′-AGGCCCAAGCCAATAGACAA-3′	5′-CCAAGAGCATTTTCCAACGC-3′	203
DS2831	5′- CGGGTATGAGACCGCAAGAA -3′	5′- GCCGCCCAAGGATAAGACAT-3′	417
	5′ GAATCATTGAGCGCCAGCAG -3′	5'-ATCCCGGTATTTCGAGCCAC-3′	163
DS0725	5′-AAGGGGGACCGATTACCTCA-3′	5′-AGTGCGGCGGAAAGAACTAA-3′	557
	5′-CGCTCAGCCCGAAGTAGAAT-3′	5′-GACGACTGAACCAACGGCTA-3′	133
DS2766	5′-CCGGCATAATGTTGGCTTCG-3′	5′-GGCAATCGCTGCATTATGGG-3′	358
	5′-GCAGAACAACATGTGCCTCA-3′	5′-TACTTTGGTGTGCCGTGGAG-3′	116
DS1566	5′-AATCAAAGCCTGCTACGCCT-3′	5′-TACGGGCTGTCCAAATGACC-3′	541
	5′-GAGGCGCGTCAGAAAATCAC-3′	5′-TTTCCGCTCGGTCAATGTCA-3′	138

Table S3. EFSA guideline for resistant Microbiological cut-off values (mg/L).

	ampicillin	vancomycin	gentamycin	kanamycin	streptomycin	erythromycin	clindamycin	tetracycline	chloramphenicol
Lactobacillus obligate homofermentative[a]	1	2	16	16	16	1	1	4	4
Lactobacillus acidophilus group	1	2	16	64	16	1	1	4	4
Lactobacillus obligate heterofermentative[b]	2	n.r.	16	32	64	1	1	8	4
Lactobacillus reuteri	2	n.r.	8	64	64	1	1	16	4
Lactobacillus facultative heterofermentative[c]	4	n.r.	16	64	64	1	1	8	4
Lactobacillus plantarum/pentosus	2	n.r.	16	64	n.r.	1	2	32	8
Lactobacillus rhamnosus	4	n.r.	16	64	32	1	1	8	4
Lactobacillus casei /paracasei	4	n.r.	32	64	64	1	1	4	4
Bifidobacterium	2	2	64	n.r.	128	1	1	8	4
Pediococcus	4	n.r.	16	64	64	1	1	8	4
Leuconostoc	2	n.r.	16	16	64	1	1	8	4
Lactococcus lactis	2	4	32	64	32	1	1	4	8
Streptococcus thermophilus	2	4	32	64	64	2	2	4	4
Bacillus spp	n.r.	4	4	8	8	4	4	8	8
Propionibacterium	2	4	64	64	64	0.5	0.25	2	2
Other Gram +	1	2	4	16	8	0.5	0.25	2	2

n.r. not required.
[a] including *L. delbrueckii, L. helveticus*
[b]including *L. fermentum*
[c]including the homofermentative species *L. salivarius*

Fig. S1. PCR confirmation of plasmid recovery in clones derived from the re-cultivation of DS1989 colonies with two or more cured plasmids after initial curing. **(A)** Results of the initial curing of DS1989. B, Results of the second curing after re-cultivation. Compared to A, plasmids 1 and 3 were recovered in lane 1, plasmid 3 in lane 2 and lane 3, plasmid 2 in lane 4, and no plasmids were recovered in lane 5.

A **B**

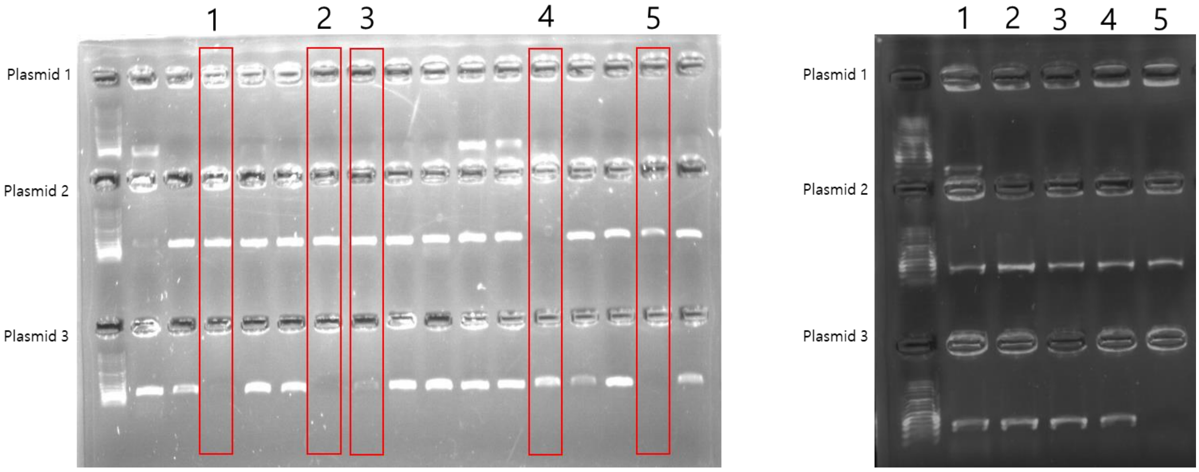

Supplement: Supplementary file 1 [file jmb-34-11-2385-supple.pdf]
